# Moderate-to-severe atopic dermatitis patients show increases in serum C-reactive protein levels, correlating with skin disease activity

**DOI:** 10.12688/f1000research.12422.2

**Published:** 2017-10-27

**Authors:** Anjali S. Vekaria, Patrick M. Brunner, Ahmad I. Aleisa, Lauren Bonomo, Mark G. Lebwohl, Ariel Israel, Emma Guttman-Yassky

**Affiliations:** 1 Department of Dermatology, Icahn School of Medicine at Mount Sinai, New York, NY, 10029, USA; 2 The Laboratory for Investigative Dermatology, The Rockefeller University, New York, NY, 10065, USA; 3 Department of Family Medicine, Clalit Health Services, Jerusalem, 954323, Israel

**Keywords:** Atopic dermatitis, C-reactive protein, systemic inflammation, disease biomarker

## Abstract

**Background**: Atopic dermatitis (AD), the most common chronic inflammatory skin disease, is evolving as a systemic disease, and associated systemic inflammation is possibly linked to increases in cardiovascular disease.

**Methods**: We assessed levels of the inflammatory marker CRP in 59 patients with moderate-to-severe AD compared to matched healthy controls, and to determine correlation with skin disease severity. Clinical severity was measured using SCORing of Atopic Dermatitis (SCORAD) and body surface area (BSA). Control subjects (n=118), matched by age, gender, smoking status and ethnicity, were obtained from the National Health and Nutrition Survey (NHANES).

**Results**: AD patients had significantly increased serum CRP levels compared to controls (0.7±1.0 vs. 0.4±0.7mg/dl; p=0.001), and 52.5% of them showed CRP levels >0.3mg/dl, predicting high cardiovascular risk. CRP levels were significantly correlated with both SCORAD (r=0.427, p=0.0008) and BSA (r=0.407, p=0.0015).  IgE levels in AD were highly elevated (median 2903U/ml, IQR [234,10655]), but only weakly correlated with SCORAD (r=0.282, p=0.0427) and BSA (r=0.382, p=0.0052), but not with CRP levels. AD patients also showed increased LDH levels, but without significant correlations with disease severity (SCORAD, BSA) or CRP.

**Conclusions**: Our study strongly supports CRP as a marker for disease severity in moderate-to-severe AD patients, further demonstrating its chronic systemic nature.

## Introduction

Atopic dermatitis (AD), the most common chronic inflammatory skin disease, frequently starts during infancy, and in adults it has usually been present for several decades ^1^. Similar to moderate-to-severe psoriasis, there is now evolving evidence that AD also has a systemic component beyond the classic atopic/allergic comorbidities, with increases in cardiovascular risk factors such as obesity ^2– 4^, and associations with cardiovascular diseases in population-based studies ^2, 5^. A comparison of AD and psoriasis patients with healthy individuals, using cardiac computed tomography angiography, showed higher rates of coronary artery disease in both psoriasis and AD, compared to controls ^6^. Systemic immune activation in adult moderate-to-severe AD patients is reflected by highly activated circulatory T-cells as measured using T-cell activation markers (ICOS and HLA-DR), at even higher frequencies than in psoriasis ^7^. Also, several inflammatory blood biomarkers (e.g. Thymus and Activation Regulated Chemokine /TARC or CCL17) were consistently shown to correlate with AD clinical severity ^8^. The important contribution of chronic inflammation to the development of atherosclerosis and cardiovascular disease events is now well established ^9^. Therefore, C-reactive protein/CRP, an acute phase reactant reflecting systemic inflammation, has been suggested as potential biomarker for cardiovascular disease ^9^. In patients with a history of myocardial infarction, the anti-inflammatory monoclonal antibody canakinumab (IL-1β blocker) led to a significant decrease in cardiovascular events ^10^. Patients also showed reductions in serum CRP levels, without changes in their lipid profile ^10^, demonstrating that anti-inflammatory treatment can indeed have an impact on cardiovascular disease. In psoriasis, it has been demonstrated that CRP is significantly elevated and associated with disease severity ^11^. One recent study suggests that CRP levels are also increased in adult chronic AD patients vs. matched controls ^12^, but it remains to be determined whether CRP could serve as a marker for disease severity. In contrast to adults, studies in children and adolescents with active AD did not show increases in overall CRP levels compared to controls ^13^, and elevated CRP levels early in life were claimed to have a protective role against the development of AD ^14^ and allergic sensitization ^15^, suggesting that chronic low-grade inflammation in infants might provide some protection from allergen sensitization. In order to better clarify the potential role of CRP as disease biomarker, we sought to investigate CRP serum levels in moderate-to-severe adult AD patients in relation to skin disease severity.

## Methods

### Study population

We retrospectively assessed CRP levels in serum from 59 adult AD patients (>18yo), with active AD and a Body Surface Area/BSA>10% (mean 59.6±27.9%, range 11–99%), that had presented to the outpatient clinic of the Department of Dermatology at Mount Sinai Hospital, New York, NY. All patients reported chronic AD since early infancy, and were off systemic anti-inflammatory AD treatment. Clinical severity was measured using SCORing of Atopic Dermatitis (SCORAD), and the vast majority of patients were in the moderate-to-severe category ^16^ (mean SCORAD 62.2±20.86, range 15–97.5). Other demographic data was also collected, including age (mean 39.5±15.2, range 18–67 years), gender (49.2/51.8% F:M), BMI (mean 27.5±5.6kg/m ^2^, range 18.99–41.62), blood pressure (mean 123.5/77.1mmHg, range systolic 80–154, diastolic 58–109), smoking status (11.9% smokers), total serum IgE (median 2903U/ml, IQR [234,10655]), ethnicity, comorbid conditions, concomitant medications and lipid profiles ( Dataset 1 ^17^). We also evaluated serum lactate dehydrogenase/LDH (mean 293.8U/L±115.3, range 117–597U/L), previously reported as a possible serum biomarker of AD severity ^18^. None of the patients showed clinical signs of active skin infection.

### Matching

Matched control subjects were obtained with a ratio of 2-to-1 (n=118) from the National Health and Nutrition Survey/NHANES ( https://www.cdc.gov/nchs/nhanes). They were matched to AD patients for age, gender, smoking status and ethnicity, using the R procedure *MatchIt*, method ‘nearest’, with a ratio of 2 control subjects for each case subject. We used individuals from the SPRINT survey nationwide between the years 2005 and 2010, for which CRP laboratory data were available. There were no changes (from the previous 2 years of NHANES) to equipment, laboratory methods or lab site.

### CRP serum level measurement

Serum CRP levels in AD patients were assessed using an immunoturbidimetric test ( *Abbott Laboratories, Lake Bluff, Illinois*). For NHANES, CRP levels were assessed using a Siemens/Behring Nephelometer ( *Siemens HealthcareDiagnostics, Deerfield, IL*), as described at https://wwwn.cdc.gov/Nchs/Nhanes/2009-2010/CRP_F.htm. Both assays had a lower limit of detection of 0.02mg/dl. While different assays were used to measure CRP levels in patients and controls, both methods have the same lower level of detection (0.02mg/dL) and were shown to be comparable ^19^.

### Statistical analysis

For comparisons between AD and the control group, we used the two sample t-test for age; Fisher exact test for gender, ethnicity and smoking status; and the two sample Wilcoxon test for biomarkers. When variables were missing for some of the individuals, comparison was performed only for the individuals for which the variable was available.

Pearson correlation coefficients were used to calculate the association between the logarithm of the biomarkers (CRP, LDH, total serum IgE) and disease activity measures SCORAD and BSA. We used a univariate linear regression formula to draw the regression line for these correlations. Each correlation was performed only for the individuals for which relevant biomarker data was available. All analyses were performed using R statistical software ( *Version 3.3*).

## Results

There were no significant differences between demographic data of AD patients and controls (age, gender, ethnicity), blood lipids (triglycerides, LDL, HDL), body mass index (BMI), or smoking status ( Table 1).

**Table 1.** Baseline characteristics and blood biomarker levels.

	Control	Atopic Dermatitis	p-value
	n= 118	n= 59	
**Age in years,** **mean (SD)**	40.3 (14.2)	39.5 (15.2)	0.707
**Female gender**	58 (49.2%)	29 (49.2%)	1.000
**Race and** **Ethnicity (%)**			0.883
Hispanic	8 (6.8%)	6 (10.2%)	0.555
Non-Hispanic White	71 (60.2%)	35 (59.3%)	1.000
Non-Hispanic Black	21 (17.8%)	9 (15.3%)	0.832
Other	18 (15.3%)	9 (15.3%)	1.000
**Smoking (%)**			1.000
Missing	4 (3.4%)	2 (3.4%)	1.000
NO	100 (84.7%)	50 (84.7%)	1.000
YES	14 (11.9%)	7 (11.9%)	1.000
**CRP mg/dL (SD)**	0.4 (0.7)	0.7 (1.0)	**0.001
**LDH U/L (SD)**	132.7 (30.3)	293.8 (115.3)	***< 0.00001
**Triglycerides** **mg/dL (SD)**	136.1 (158.3)	130.0 (69.8)	0.482
**LDL mg/dL (SD)**	116.1 (31.3)	111.5 (38.2)	0.357
**HDL mg/dL (SD)**	54.1 (14.4)	60.6 (27.8)	0.376
**Body Mass Index** **kg/m ^2^ (SD)**	28.1 (6.8)	27.5 (5.6)	0.781

Comparisons of AD patients with matched healthy controls. *Two samples t-test (age), Fisher exact test (gender, ethnicity, smoking), Wilcoxon test (CRP, LDH, triglycerides, LDL, HDL, BMI).*

**Figure 1.**
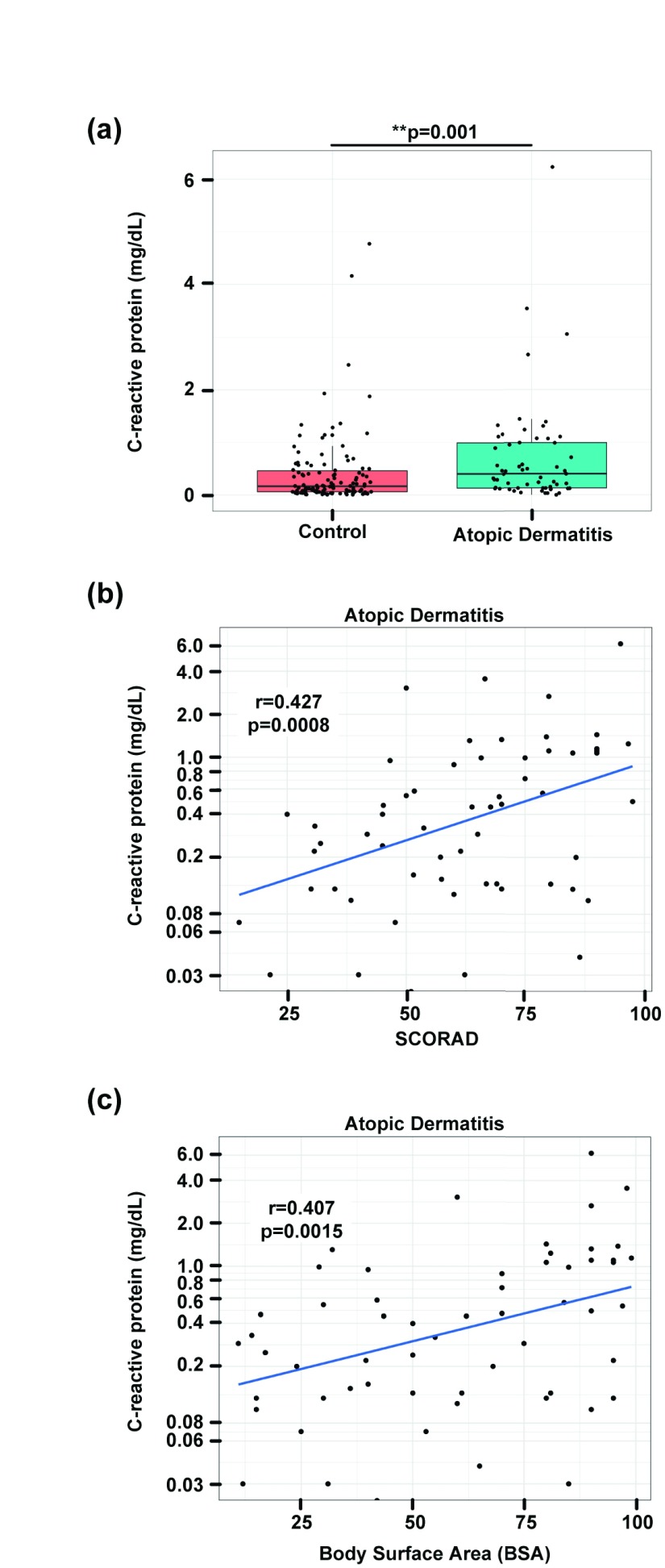
C-reactive protein levels are increased in AD patients. Comparison of CRP levels (mg/dL) in AD patients and healthy control subjects; *Wilcoxon-test: p=0.001* ( **a**); *Pearson correlation and linear regression* of CRP levels with SCORAD ( **b**) and body surface area/BSA ( **c**).

**Figure 2.**
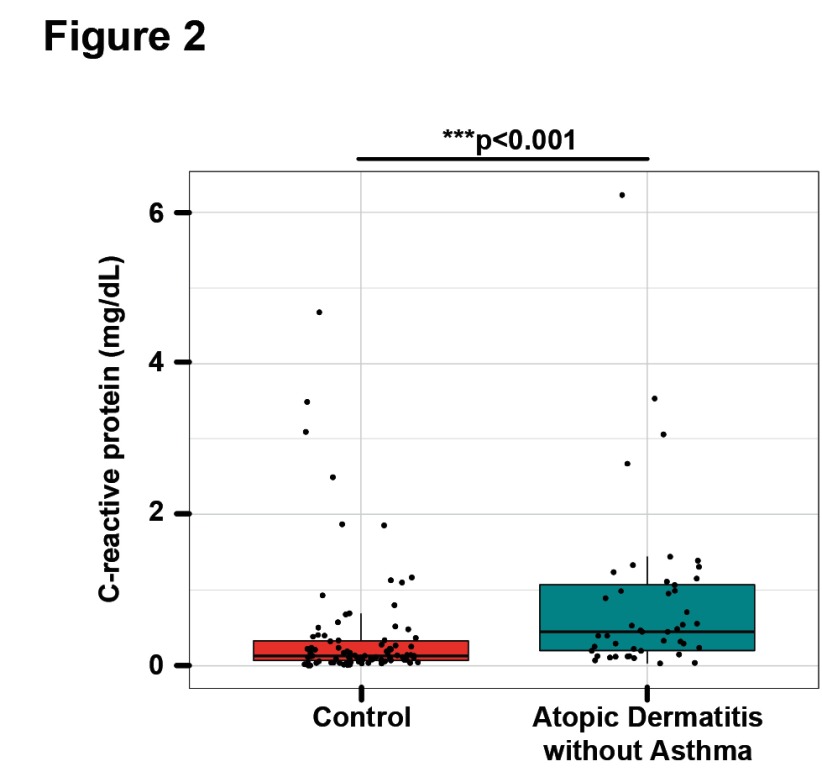
C-reactive protein levels are increased in AD patients without asthma. CRP levels (mg/dL) in AD patients excluding those with a history of asthma, compared to matched healthy control subjects; *Wilcoxon-test: p<0.001*.

AD patients had significantly increased serum CRP levels (0.7±1.0mg/dl) when compared to controls (0.4±0.7mg/dl; p=0.001; Table 1 and Figure 1a). CRP levels in AD ranged from undetectable in one patient (<0.02mg/dl) to a maximum value of 6.2mg/dl in a patient with very severe AD and a SCORAD of 95 ( Dataset 1 ^17^). 23 out of 59 patients (39%) showed CRP levels outside the reference range of 0-0.5mg/dl. Furthermore, CRP levels were significantly correlated with both SCORAD ( Figure 1b) and BSA ( Figure 1c). As 14 patients reported a history of asthma, a disease that has been shown to be associated with increased CRP blood levels ^20^, we performed a sensitivity analysis to assess the non-asthma AD patients ( Supplementary Table 1). However, differences between CRP levels in AD patients and controls remained highly significant after exclusion of all the patients with a history of asthma ( Figure 2, Supplementary Table 1).

**Figure 3.**
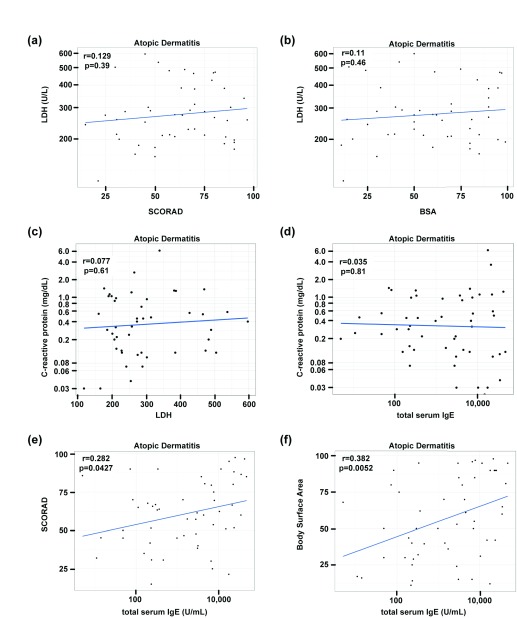
Blood biomarker and skin correlations. LDH and total serum IgE levels correlated with SCORAD, body surface area/BSA and CRP levels ( **a**– **f**); *Pearson correlation and linear regression*.

Consistent with previous publications ^18^, the AD patients also showed increased LDH levels, but without significant correlations with disease severity measures (SCORAD, BSA) or CRP ( Figure 3a–c). While IgE levels in AD were highly elevated (median 2903U/ml, IQR [234,10655]) and correlated with SCORAD and BSA, they were not correlated with CRP ( Figure 3d–f).

Individual demographics, biomarkers and comorbid conditions of the AD study patients Click here for additional data file. Copyright: © 2017 Vekaria AS et al. 2017 Data associated with the article are available under the terms of the Creative Commons Zero "No rights reserved" data waiver (CC0 1.0 Public domain dedication).

## Discussion

This study is the first to demonstrate a correlation of AD disease severity with CRP levels in moderate-to-severe adult AD patients with decades of chronic disease activity, independent of co-existence of asthma. This finding is in line with the evolving concept that chronic AD has a considerable systemic inflammatory component ^12^ that is directly linked with the overall inflammatory burden in the skin. This increase in systemic inflammation might not only be a biomarker for skin disease severity, but one might speculate that it could also contribute to AD comorbid conditions, such as the evolvement of cardiovascular disease ^2^. This concept is strongly supported by the fact that canakinumab led to a significant reduction in serum CRP levels and cardiovascular events in a recent clinical trial ^10^. Interestingly, a case series using the IL-6R blocker tocilizumab was efficacious in AD, and decreased CRP levels, but was not followed further due to bacterial superinfection ^21^. According to the joint guidelines of the Centers for Disease Control and Prevention and the American Heart Association on CRP levels and cardiovascular risk ^11^, 20 of our AD patients (33.9%) showed CRP levels in the range of ≥0.1mg/dl and ≤0.3mg/dl, predicting intermediate risk, and 31 patients (52.5%) showed CRP levels >0.3mg/dl, which is within the high risk range. While CRP is predominantly produced by hepatocytes, it has also been detected in tape stripping experiments from AD skin, and its expression responded to emollients ^22^.

Future large and prospective studies in chronic severe AD patients should determine whether the up-regulated CRP levels observed in our AD cohort are indeed linked to increased cardiovascular risk, beyond its role as a marker of systemic inflammation. Nevertheless, there is some circumstantial evidence that even these small increases might be clinically relevant, as CRP above 0.42mg/dL showed differences in statin treatment outcomes for cardiovascular events in a clinical trial ^23^, and CRP levels in the canakinumab trial were in the same order of magnitude ^10^.

Future clinical trials investigating new therapeutic agents might follow changes in CRP levels during treatment as a potential serum biomarker of disease severity and systemic inflammation, and these may clarify whether correcting CRP can serve as a surrogate for decreasing cardiovascular risk in AD patients. However, increases in CRP levels can be a result of various conditions such as infections and malignancies, which needs to be taken into account.

Our study harbors a few limitations. Besides being a retrospective study, healthy controls were not available at our site and were based on published historic controls matched for age, gender, and ethnicity. Also, it focused on a moderate-to-severe AD patient population (all but two patients had moderate-to-severe AD, i.e. a SCORAD >25 ^16^) in a large tertiary academic center in New York, while controls were obtained across the United States, which might introduce some bias. To ensure that our results are applicable to the general AD population across ethnicities, larger international studies across different ethnic backgrounds that will also evaluate for existence of “silent” cardiovascular disease in chronic AD patients are needed. However, our data supports the role that persistent skin disease has in the systemic burden of inflammation in AD patients, mandating further investigation.

## Ethical statement

This study has been approved by the IRB of the Icahn School of Medicine at Mount Sinai, New York, NY (approval number, 16-00717), according to the Declaration of Helsinki.

## Data availability

The data referenced by this article are under copyright with the following copyright statement: Copyright: © 2017 Vekaria AS et al.

Data associated with the article are available under the terms of the Creative Commons Zero "No rights reserved" data waiver (CC0 1.0 Public domain dedication). 

Dataset 1: Individual demographics, biomarkers and comorbid conditions of the AD study patients. doi, 10.5256/f1000research.12422.d177784 ^17^

## Supplementary material

**Supplementary Table 1. Baseline characteristics and blood biomarker levels of AD subset without asthma.** AD patients excluding those with a history of asthma, compared with matched healthy controls. *Two samples t-test (age), Fisher exact test (gender, ethnicity, smoking), Wilcoxon test (CRP, LDH, triglycerides, LDL, HDL, BMI)*.
