# Supplementary material for: Moderate-to-severe atopic dermatitis patients show increases in serum C-reactive protein levels, correlating with skin disease activity
Source: F1000Res. 2017 Oct 27;6:1712. Originally published 2017 Sep 20. [Version 2] doi: 10.12688/f1000research.12422.2 (PMC5698919; doi:10.12688/f1000research.12422.2)
Supplement: Supplementary file 2 [file f1000research-6-14051-s0001.tgz › 556397ff-51bd-42dc-88da-4b2e8c51beaf.docx]

**Supplementary Table 2.** Demographic and biomarker data of the AD patients investigated

|  | **Control** | **Atopic Dermatitis**  **Without Asthma** | **p-value** |
| --- | --- | --- | --- |
|  | n=90 | n=45 |  |
| **Age in years, mean (SD)** | 44.7 (14.6) | 43.0 (14.4) | 0.539 |
| **Female gender** | 39 (43.3%) | 20 (44.4%) | 1.000 |
|  |  |  |  |
| **Race and Ethnicity (%)** |  |  | 0.982 |
| Hispanic | 8 (8.9%) | 5 (11.1%) | 0.759 |
| Non-Hispanic White | 50 (55.6%) | 25 (55.6%) | 1.000 |
| Non-Hispanic Black | 14 (15.6%) | 7 (15.6%) | 1.000 |
| Other | 18 (20.0%) | 8 (17.8%) | 0.821 |
|  |  |  |  |
| **Smoking (%)** |  |  | 1.000 |
| Missing | 4 (4.4%) | 2 (4.4%) | 1.000 |
| NO | 72 (80.0%) | 36 (80.0%) | 1.000 |
| YES | 14 (15.6%) | 7 (15.6%) | 1.000 |
|  |  |  |  |
| **CRP mg/dL (SD)** | 0.4 (0.8) | 0.8 (1.1) | ***< 0.00001 |
| **LDH U/L (SD)** | 140.4 (38.1) | 291.8 (110.8) | ***< 0.00001 |
| **Triglycerides mg/dL (SD)** | 142.0 (64.1) | 134.7 (67.9) | 0.467 |
| **LDL mg/dL (SD)** | 112.9 (28.2) | 115.2 (39.7) | 0.811 |
| **HDL mg/dL (SD)** | 53.7 (15.8) | 60.7 (30.1) | 0.395 |
| **Body Mass Index kg/m^2^ (SD)** | 27.4 (5.2) | 28.0 (5.8) | 0.627 |

**Supplementary Table 2.** **Baseline characteristics and blood biomarker levels of AD subset without asthma.** AD patients excluding those with a history of asthma, compared with matched healthy controls. *Two samples t-test (age), Fisher exact test (gender, ethnicity, smoking), Wilcoxon test (CRP, LDH, triglycerides, LDL, HDL, BMI).*
