# Supplementary material for: Moderate-to-severe atopic dermatitis patients show increases in serum C-reactive protein levels, correlating with skin disease activity
Source: F1000Res. 2017 Oct 27;6:1712. Originally published 2017 Sep 20. [Version 2] doi: 10.12688/f1000research.12422.2 (PMC5698919; doi:10.12688/f1000research.12422.2)
Supplement: Peer review history

# Peer review: Referee response for version 2

**DOI:** 10.5256/f1000research.14051.r27679

**Published:** 2017-11-21

**Authors:** Alan Menter, Isabel Haugh

**Affiliations:** 1 Division of Dermatology, Baylor University Medical Center, Dallas, TX, USA; 2 Baylor Dermatology Residency Program, Baylor University Medical Center, Dallas, Texas, USA

This is a quality and important review by Vekaria et al from the Dermatology program of Mount Sinai in New York with Emma Guttman-Yassky- a leading clinician and researcher in the field of Atopic Dermatitis worldwide as corresponding author. 

 The nature of Atopic Dermatitis as a systemic inflammatory disease with comorbidities has been significantly accelerated over the past five years, especially with the initiation of the International Eczema Council (IEC) by Dr.s Guttman-Yassky and Paller. Of interest are the 3 meetings the IEC has had with the International Psoriasis Council (IPC) comparing the imuunopathogenesis and comorbidities of Atopic Dermatitis and Psoriasis. 

 Of interest, in this C-reactive protein review of 59 patients in Atopic Dermatitis Reference #11 (B. Strober et al ^1^) mentions the increased levels of CRP in psoriasis. It is important to recognize now that psoriasis has been demonstrated to be a systemic immune-mediated disease that CRP levels in psoriasis patients with moderate to severe disease are significantly lower than in psoriasis patients who develop Psoriatic Arthritis and also are lower than other immune-mediated systemic disease, e.g. Crohn's or Rheumatoid Arthritis. 

 We do believe it should be emphasized in the title and abstract that this CRP review was carved out in adults only. In addition, it should be clarified in the method section that data was obtained retrospectively. 

 We all recognize that CRP is an acute phase reactant that can increase with infections/autoimmune/cancer as well as cardiovascular disease. Thus, reference should be made in this article to comorbidities in this group of 59 Atopic Dermatitis patients which could have possibly played a role in the increases of CRP.

We have read this submission. We believe that we have an appropriate level of expertise to confirm that it is of an acceptable scientific standard.

## References

[1] StroberBTellerCYamauchiPMillerJLHooperMYangYCDannF: Effects of etanercept on C-reactive protein levels in psoriasis and psoriatic arthritis. *Br J Dermatol*.2008;159(2) : 10.1111/j.1365-2133.2008.08628.x 322-30 10.1111/j.1365-2133.2008.08628.x 18503600

# Peer review: Referee response for version 2

**DOI:** 10.5256/f1000research.14051.r27404

**Published:** 2017-11-20

**Authors:** Alexander A. Navarini

**Affiliations:** 1 Department of Dermatology, University Hospital of Zurich, Zurich, CH-8091, Switzerland

Thank you, my concerns have now been fully addressed.

I have read this submission. I believe that I have an appropriate level of expertise to confirm that it is of an acceptable scientific standard.

# Peer review: Referee response for version 2

**DOI:** 10.5256/f1000research.14051.r27475

**Published:** 2017-11-06

**Authors:** John C. Su

**Affiliations:** 1 Department of Dermatology, Eastern Health Clinical School, Monash University, Melbourne, Vic, Australia

1. This is an interesting and considered study, a single-centre series of 59 adult patients matched 1:2 with controls from the nationwide NHANES survey. CRP, LDH and sIgE were correlated with AD severity, assessed by SCORAD and BSA.

 Was there a reason for choosing SCORAD over objective SCORAD or EASI, which rely only on objective measures? Of interest, was CRP in adult AD vs non-AD using the NHANES data itself done (cf Silverberg Pediatr Allergy Immunol 2015)?

 2. CRP, a marker of cardiovascular risk, was found to be elevated in AD, and correlated with AD severity. sIgE correlated with AD severity, but not with CRP. LDH did not show significant correlation with AD severity or CRP.

 In addition to the Silverberg pediatric paper, Park et al ^1^ presented a pediatric inpatient study of 67 children examining the relationship between eczema severity (mild-moderate vs severe) and a number of laboratory markers. They did not find correlation with CRP, LDH or skin cultures. That paper was pediatric, written in Korean and also has limitations, but may be worth referencing for comparison.  

 3. Some potential confounders were considered. Sensitivity analysis was performed for non-asthma AD patients; this subgroup still showed correlation between CRP and AD severity. The patients did not have clinical infection. Were any swabs done? Some patients in the dataset had other co-morbidities that could have contributed to the CRP. The nature of the control group may not allow ready comparison of these, but a comment about possible or unlikely confounding from co-morbidities as the case may be worth considering.

I have read this submission. I believe that I have an appropriate level of expertise to confirm that it is of an acceptable scientific standard.

## References

[1] ParkGParkJHwangYSungMKimS: The correlation between the severity of atopic dermatitis classified by SCORing atopic dermatitis index and the laboratory tests. *Allergy Asthma & Respiratory Disease*.2013;1(1) : 10.4168/aard.2013.1.1.79 10.4168/aard.2013.1.1.79

# Peer review: Referee response for version 1

**DOI:** 10.5256/f1000research.13452.r26213

**Published:** 2017-10-11

**Authors:** Alexander A. Navarini

**Affiliations:** 1 Department of Dermatology, University Hospital of Zurich, Zurich, CH-8091, Switzerland

Vekaria, Brunner *et al.* present a nice and clear clinical Investigation into AD severity and CRP levels.

 The title should be adapted to "Moderate-to-severe AD patients ..." as you have really investigated just this population.  

 In the correlations, the patients with high CRP Levels are omitted for some (graphical?) reason. Please state why and whether the Pearson r is calculated with or without them. I don't think this changes the conclusion of the paper but IMHO should be shown. If you have access to the raw SCORAD data, you might be able to check whether subcomponents of the SCORAD have a closer connection to the Serum CRP than others:

 - eczema involvement of some body regions

 - crusting, oozing

 - excoriations (scratch marks)

 I think it may be worth adding to the abstract that >50% of the moderate-to-severe AD patients were in the range of cardiovascular high-risk CRP levels. Also, you should probably discuss all ways to lower the high CRP. The best of them may be anti-IL6R, which also works in atopic dermatitis according to a case series.

I have read this submission. I believe that I have an appropriate level of expertise to confirm that it is of an acceptable scientific standard, however I have significant reservations, as outlined above.

# Response

**Published:** 2017-10-17

**Authors:** Emma Guttman

**Affiliations:** Icahn School of Medicine at Mount Sinai, USA

We thank the reviewer for his positive and encouraging comments. We have changed the title accordingly.

 All patients have been included in the graphs. We have now corrected the axis labeling for a more clear display of CRP levels in the correlation graphs Figures 1b and 1c, and Figures 3c and 3d.

 Due to the retrospective nature of the study, we do not have access to the sub-components of SCORAD. Therefore, we cannot calculate these correlations.

 We have now modified the abstract and the discussion section accordingly.
